# Challenges in Contemporary Spine Surgery: A Comprehensive Review of Surgical, Technological, and Patient-Specific Issues

**DOI:** 10.3390/jcm13185460

**Published:** 2024-09-14

**Authors:** Emmanuel O. Mensah, Joshua I. Chalif, Jessica G. Baker, Eric Chalif, Jason Biundo, Michael W. Groff

**Affiliations:** 1 Department of Neurosurgery, Brigham and Women’s Hospital and Harvard Medical School, Boston, MA 02115, USA; emensah2@bwh.harvard.edu (E.O.M.); chalif@bwh.harvard.edu (J.I.C.); echalif@mgb.org (E.C.); 2 Department of Behavioral Neuroscience, Northeastern University, Boston, MA 02115, USA; baker.jes@northeastern.edu; 3 F.M. Kirby Neurobiology Center, Boston Children’s Hospital, Boston, MA 02115, USA; jason.biundo@childrens.harvard.edu

**Keywords:** spine surgery challenges, spinal anatomy complexity, minimally invasive surgery, technological advancements, artificial intelligence in spine surgery

## Abstract

Spine surgery has significantly progressed due to innovations in surgical techniques, technology, and a deeper understanding of spinal pathology. However, numerous challenges persist, complicating successful outcomes. Anatomical intricacies at transitional junctions demand precise surgical expertise to avoid complications. Technical challenges, such as underestimation of the density of fixed vertebrae, individual vertebral characteristics, and the angle of pedicle inclination, pose additional risks during surgery. Patient anatomical variability and prior surgeries add layers of difficulty, often necessitating thorough pre- and intraoperative planning. Technological challenges involve the integration of artificial intelligence (AI) and advanced visualization systems. AI offers predictive capabilities but is limited by the need for large, high-quality datasets and the “black box” nature of machine learning models, which complicates clinical decision making. Visualization technologies like augmented reality and robotic surgery enhance precision but come with operational and cost-related hurdles. Patient-specific challenges include managing postoperative complications such as adjacent segment disease, hardware failure, and neurological deficits. Effective patient outcome measurement is critical, yet existing metrics often fail to capture the full scope of patient experiences. Proper patient selection for procedures is essential to minimize risks and improve outcomes, but criteria can be inconsistent and complex. There is the need for continued technological innovation, improved patient-specific outcome measures, and enhanced surgical education through simulation-based training. Integrating AI in preoperative planning and developing comprehensive databases for spinal pathologies can aid in creating more accurate, generalizable models. A holistic approach that combines technological advancements with personalized patient care and ongoing education is essential for addressing these challenges and improving spine surgery outcomes.

## 1. Introduction

Spine surgery has experienced significant advancements over the past few decades, driven by innovations in surgical techniques, technological advancements, and a deeper understanding of spinal pathology [1,2]. Despite these strides, surgeons continue to confront a wide array of challenges that complicate the path to successful outcomes. This article seeks to provide a comprehensive review of the current challenges in spine surgery, categorizing them into surgical, technological, and patient-specific issues.

Anatomical problems are among the most critical challenges in spine surgery. A deep understanding of anatomical variants, as well as anatomical orientation challenges, particularly with minimally invasive methods involving small surgical corridors, is essential. For example, the transitional anatomy of the L5/S1 junction often dictates the choice of patients for endoscopic approaches. Additionally, previous spine surgeries can deform anatomical features, complicating anatomical localization and surgical planning. These complexities demand precise surgical expertise and thorough pre- and intraoperative planning.

Technological challenges, such as the integration of artificial intelligence (AI) and advanced visualization systems, are increasingly relevant in modern spine surgery. While AI offers predictive capabilities, it is limited by the need for large, high-quality datasets and the “black-box” nature of machine learning models, which complicates clinical decision making. Visualization technologies, including augmented reality and robotic surgery, enhance precision but come with operational and cost-related hurdles.

Patient-specific challenges include managing postoperative complications such as adjacent segment disease, hardware failure, and neurological deficits. The intricacies of patient selection are essential to minimize risks and improve outcomes, yet criteria can be inconsistent and complex.

Given the multifaceted nature of these challenges, this review will highlight the importance of continued technological innovation, improved patient-specific outcome measures, and enhanced surgical education through simulation-based training.

## 2. Surgical Challenges

### 2.1. Complexity of the Spinal Anatomy

The intricate anatomy of the spinal cord presents a formidable challenge to spine surgery. Surgical intervention at different levels of the spinal cord requires complete mastery of local anatomy, in addition to thorough preoperative imaging, to prevent morbidity and treatment failure following surgery (Table 1). The craniocervical junction (CCJ), also known as the craniovertebral junction (CVJ), separates the cranium from the cervical spine. It represents a complex transitional zone that is anatomically distinct from both the cranium and the cervical spine [3]. Comprising osseous structures of the occiput and first two cervical vertebrae, the CCJ houses the spinal cord, the lower cranial nerves (IX–XII), and is closely related to the vertebral artery [3,4]. In addition, limited anatomic landmarks and restricted visualization of this region with the use of current technologies increase the risk of complications following spinal interventions. Instrumentation techniques of the cervical spine, given its close apposition to the vertebral artery, offer a low margin of error, especially in individuals with anatomic variants and previous cervical surgery [5]. The complex anatomy of the cervical spine has been demonstrated to dictate surgical approaches. For example, posterior subtraction osteotomies for cervical spine deformities are largely performed at the C7 level due to its spacious canal and the more cranial course of the vertebral artery [5]. The cervicothoracic junction (CTJ) also presents unique anatomical and mechanical challenges. Instrumentation of the CTJ demands specific and tailored techniques due to its transitional nature between the cervical subaxial and thoracic spine. The CTJ is characterized by thin and convergent pedicles, requiring surgeons to carefully consider the extent of arthrodesis to prevent instrumentation failure and adjacent segment disease [6]. The thoracic spine, in comparison to the lumbar spine, exhibits smaller pedicle sizes, which increase the likelihood for spinal cord injury following instrumentation techniques, such as the placement of percutaneous screws [7,8]. Similarly to the CVJ, the transitional anatomy of the lumbosacral junction (L5-S1) presents challenges with regard to surgical access and limited visualization. Treatment of disc herniations at this site with minimally invasive techniques is hindered by a large sacral ala, large L5 transverse processes, a high-riding ilium, and a lateral-lying lumbosacral facet joint [9]. Thus, surgeries in this region may require larger surgical corridors to mitigate complications of nerve root trauma associated with the blind removal of residual intraforaminal disc fragments [10,11].

### 2.2. Variability in Patient Anatomy

In addition to navigating the complex anatomy of the spine, the spine surgeon also requires a thorough understanding of anatomic variants that may increase the morbidity likelihood of surgical intervention, including surgery at the wrong level. Anatomic variants at the transitional junctions have been reported in the literature. CCJ anomalies include atlantooccipital assimilation and basilar invagination. Atlantooccipital assimilation refers to the partial or complete lack of segmentation between the skull and the first cervical vertebra, potentially accompanied by fusion of the second and third cervical vertebrae [12,13]. Basilar invagination involves encroachment of the odontoid process into the foramen magnum, commonly seen in conditions like rheumatoid arthritis, Klippel–Feil syndrome, and Chiari malformation [12,14]. Anatomic variations in the cervical spine, including high-riding vertebral arteries, C1 lateral mass dysplasia, and narrow C2 pedicles, may increase morbidity in surgery at the CCJ [15]. Thoracolumbar transitional vertebrae, characterized by features of both thoracic and lumbar vertebrae at the thoracolumbar junction, and lumbosacral transitional vertebrae, defined by the sacralization of lumbar vertebrae or lumbarization of sacral vertebrae, pose challenges in spinal enumeration [16,17,18].

Rib variants may also lead to wrong entry points for spinal interventions. Cervical ribs, typically arising from the seventh cervical vertebra, may be complete (articulating with the first rib) or incomplete (freely ending in the neck’s soft tissues), leading to inaccurate identification of spinal levels during surgery [19,20]. In the thoracic spine, rib variants may be structural or numerical, including bifid, fusion, and hypoplastic ribs [19]. A less common rib variant is the lumbar rib, which is an additional rib that resembles normal ribs but appears to be floating and follows a different course [19,21].

Hemivertebrae refers to a condition where only half of the vertebral body develops, leading to spinal curvature and complicating intraoperative-level identification. Hemivertebrae are more prevalent in the thoracic spine but can also occur in the cervical and lumbar regions [22]. Once properly identified, however, hemivertebrae may serve as beneficial landmarks during surgery to mitigate the risk of operating at the wrong level. Block vertebrae represent another variation in normal spinal anatomy. This can be a congenital fusion of adjacent vertebrae, primarily observed in the cervical and lumbar regions, or acquired fused vertebrae, characterized by their lower height and lack of a radiological “waist” [23,24].

In addition to the anatomic variants, previous spine surgery may deform anatomical features, lead to scar formation, and introduce instrumentation, obliterating anatomic landmarks and complicating anatomic localization [22]. Detailed surgical planning and comprehensive pre- and intraoperative imaging is critical for accurate spinal enumeration, ultimately leading to safer surgical outcomes.

### 2.3. Technical Complexity of Modern Approaches

Modern approaches in spine surgery boast many advantages. However, they do not come without demerits, notably the learning curve associated with their performance. Minimally invasive techniques offer a small surgical corridor, presenting challenges in anatomic orientation, hand–eye coordination, and dexterous manipulation of instruments. These challenges are well documented in endoscopic spine surgery, where challenges with the understanding and visualization of spinal anatomy are met with procedural difficulty. Lewandroski et al. [25], with this consideration, list a number of endoscopic spinal surgery interventions that a beginner surgeon must avoid, including challenging disc herniations and facet cysts. Endoscopic spine procedures may also cause adverse effects due to incorrect manipulation of equipment, such as penetration of the anterior annulus from plunging the guidewire too deeply, or slippage of trephines and reamers from the target area [25].

Endoscopic spine procedures are increasingly being adopted due to their minimally invasive nature and ability to preserve surrounding tissues. However, these techniques present their own set of challenges, particularly when it comes to differential diagnosis and precision in targeting specific pathologies. For instance, spontaneous resolution of symptomatic synovial cysts, as outlined by recent studies [26], highlights the importance of thorough preoperative diagnostics to determine the need for surgical intervention. Similarly, the use of the transorbital approach, which has shown promise in multiportal variants, offers new avenues for endoscopic access while emphasizing the need for precise anatomic understanding [27].

**Figure 1:**
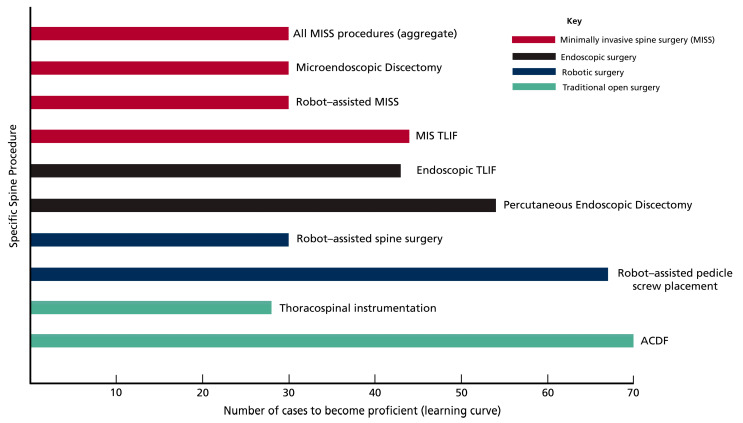
The learning curve associated with operative spinal procedures. For most MISS techniques, the learning curve to overcome operative times and complications is 20 to 30 cases [32]. Learning curves for specific MISS interventions range from 20 to 44 cases for MIS-TLIF [36,37,38] and 25–30 for microendoscopic discectomy [39,40]. For endoscopic procedures, they range from 34 to 41 cases for endoscopic TLIF [41,42] and 18 to 54 cases for percutaneous endoscopic discectomy [43,44]. Robot-assisted spine surgeries require a learning curve of 14 to 30 cases overall for robot-assisted spine surgery [45,46,47] and 13 to 67 cases for robot-assisted screw placement [47,48,49,50,51]. For open spinal procedures, learning curves of 28 cases for thoracospinal instrumentation [52] and 70 cases for ACDF [29] are reported.

To provide quantitative context of the learning curve with endoscopic lumbar spine procedures, a spine surgeon must perform between 15 and 80 cases to master the skill [28]. The number of cases required to achieve competency is also reported for other spinal interventions; this ranges from 17 to 57 cases for anterior cervical discectomy and fusion [29], around 25 cases for augmented reality/virtual reality technologies [30], and between 17 and 52 cases for freehand placement of thoracolumbar pedicle screws [31]. A systematic review assessing the learning curve associated with minimally invasive approaches to spinal decompression and fusion reported that it requires performing between 20 and 30 cases to reduce the operative time and complications for spine interventions, including transforaminal lumbar interbody fusion (TLIF), minimally invasive lumbar decompression, percutaneous pedicle screw insertion, laparoscopic anterior lumbar interbody fusion (ALIF), and minimally invasive cervical procedures [32] (Figure 1). It should be noted that studies vary on the definition of the learning curve in spine surgery. Example metrics of overcoming this learning curve include the number of cases required to reduce estimated blood loss, fluoroscopy time, and length of postoperative stay [33]; the number of cases required to achieve surgical dexterity [34]; and the number of cases required to reduce the risk of specific complications, such as the rate of disc re-herniation after tubular microdiscectomy [35].

## 3. Technological Challenges

### 3.1. Artificial Intelligence Systems

**Figure 2:**
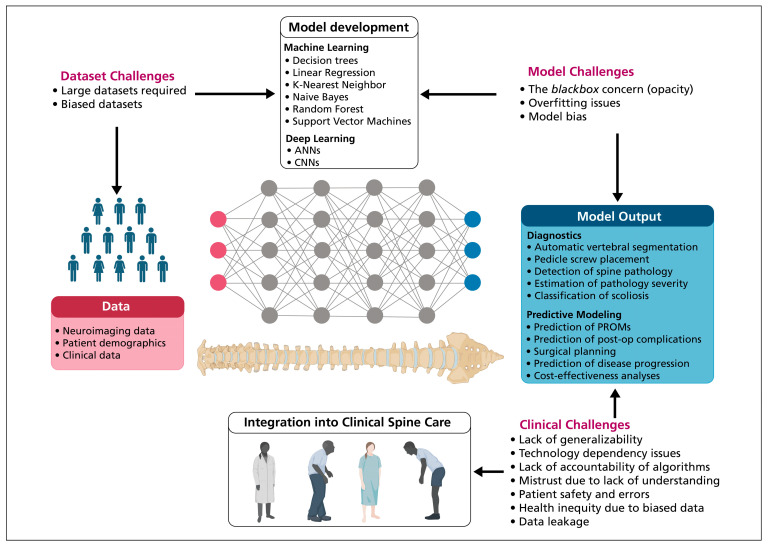
Challenges of the use of AI algorithms in spine surgery. Integration of AI systems into spine surgery workflow is limited by dataset challenges, inherent model challenges, and clinical challenges.

Artificial intelligence (AI) systems, particularly those utilizing machine learning (ML) and neural networks, have been increasingly integrated into spine surgery with the aim of enhancing patient outcomes, predicting surgical site infections, and improving the accuracy of surgical interventions [53,54,55]. Despite its potential, the application of AI in spine surgery is fraught with limitations and ethical challenges that necessitate careful consideration (Figure 2). The application of AI to spine surgery requires substantial patient datasets, which are heavily relied upon by ML systems, such as artificial neural networks (ANNs), logistic regression (LR), random forest decision trees (RF), and support vector machines (SVMs) to provide accurate predictions [56]. However, SVMs struggle with datasets where the number of observations significantly exceeds the features, and their efficiency is reduced with very large datasets due to increased noise and outliers [57]. The development of truly generalizable applications for spine surgery is also severely hindered by the limited availability of large datasets dedicated to neuroimaging, which are important for covering the characteristics of spinal pathologies [58].

Another significant limitation is the “black box” nature of ML algorithms, which refers to the difficulty in interpreting how these systems reach their conclusions [59,60]. This opacity is particularly problematic in the clinical context, where understanding the rationale behind a diagnostic or prognostic assessment is crucial. The black box issue raises ethical concerns regarding patient safety. For instance, Hopkins et al. [54] found that their ANN model, despite its reliability in predicting surgical site infections, predicted a negative correlation between the Charlson Comorbidity Index and surgical site infections following posterior spinal fusions. These findings contradicted the established medical literature and highlighted the unpredictability and potential misalignment of AI models within clinical settings.

Artificial intelligence systems function on “ground truths”, which are typically provided by a human expert, and their predictive power is dictated by the quality of the data on which they are trained [58]. Distributional shift may occur with AI systems, whereby training of AI systems is performed on biased datasets. The widespread adoption of AI systems in spine surgery is further complicated by their insensitivity to the impact of false positives or negatives, their ability to reinforce outcomes they are designed to detect by “cheating the system” [61], and ethical implications of technology dependency, patient privacy, and health inequity. The integration of AI into clinical practice necessitates a collaborative approach that leverages the strengths of these technologies while mitigating their limitations to ensure that patient care remains at the forefront of surgical innovation.

### 3.2. Visualization Systems

The advent of visualization and navigation systems in contemporary spine surgery, including augmented reality (AR)/virtual reality (VR), computer-assisted navigation, computer vision technology, exoscopes, and robotic surgery, has transformed the operating room. The exoscope, in particular, has gained popularity due to its ergonomic benefits, offering improved surgeon posture during procedures, which reduces the risk of musculoskeletal disorders commonly associated with traditional operating microscopes (OMs) [62,63]. Studies have shown that the exoscope allows surgeons to maintain a neural static posture during spine surgeries, potentially reducing surgeon fatigue and improving overall comfort during long procedures [63]. Moreover, operating room staff have reported improved visualization of the surgical site when using the exoscope compared to traditional OMs [62].

However, despite these benefits, the exoscope is not without its limitations. Decreased depth perception, particularly at higher magnifications, and reduced image sharpness have been reported in ACDFs and posterior decompression procedures, especially in more complex surgeries [62,64]. Additionally, there are operational challenges, including discomfort with wearing 3D glasses, huge startup costs, battery life limitations, learning curve, and the lack of comprehensive data evaluating their safety and efficacy, associated with these technologies [30]. Addressing these challenges requires concerted efforts in innovation, standardization, and research to fully realize the benefits of these technologies in enhancing surgical precision and patient outcomes in spine surgery.

## 4. Patient-Specific Challenges

### 4.1. Complications of Spine Surgery

Spine surgery is associated with postoperative complications, with potential for significant morbidity and impact on quality of life. These complications range from minor events, which may only present on radiological imaging or as time-limited adverse events, to major complications, presenting with neurological deficit, pain, and deformity, requiring reoperation [65]. With reported complication rates of spine surgery ranging from 7 to 49% depending on the type of surgery [66,67,68], these complications can be broadly categorized as those related to spinal instrumentation and those unrelated to instrumentation (Table 2).

Complications of spine surgery related to instrumentation include dural tears, adjacent segment disease (ASD), hardware failure, junctional kyphosis, and pseudoarthrosis. Incidental dural tears occur in spine surgery due to laceration by surgical equipment, the most common of which is the Kerrison punch [69]. Revision surgery carries the highest risk for dural tears, in addition to patient-specific risk factors, such as older age, rheumatoid arthritis, and procedure-specific risk factors, including multilevel fusions and cervical spine surgeries [70,71]. Repair of dural tears is necessary to prevent CSF leaks, with direct suturing and application of glue, sealant, or graft material being strategies utilized to promote repair [70,72]. Adjacent segment pathology refers to degeneration at a vertebral level adjacent to a previously fused spine, termed adjacent segment degeneration if only radiographic signs are present, and adjacent segment disease if clinical symptoms present with radiographic signs [73]. Pathologic processes driving adjacent segment pathology include motion segment instability and abnormal postoperative sagittal alignment, leading to increased mechanical stress at adjacent levels following spinal fusion. The annual incidence of ASD has been reported to be 2.9% in the cervical spine [74] and 2.5% in the lumbar spine [75], with ASDs accounting for as much as 54% of all spinal reoperations [76]. Abnormal spinal alignment also predisposes to hardware failure, which refers to the failure of spinal instrumentation to stabilize the spine in the goal of achieving arthrodesis. Types of failure reported include rod fracture, screw breakage, and screw loosening/pullout, which may require revision surgery [77]. Rates of hardware failure in spine surgery range from 2.8% in posterolateral decompression techniques [78] to 32% in 3-column osteotomies [79], reflecting differences in the extent of instrumentation and reconstruction techniques. Increased mechanical stress in adjacent vertebrae can lead to radiographic kyphotic deformities at either end of an instrumented spinal fusion, termed proximal junctional kyphosis (PJK) if occurring at one level superior to the upper instrumented vertebra (UIV) or distal junctional kyphosis (DJK) if occurring at two levels inferior to the lowest instrumented vertebra (LIV) [66]. Diagnosis of junctional kyphosis is made by sagittal Cobb angle measurements, with most experts using a kyphosis angle of 10 degrees as a threshold [80]. Incidence rates of junctional kyphosis differ for PJK and DJK, with PJK rates ranging from 5% to 46% and DKJ rates ranging from 7.1% to 24% [81,82,83]. Pseudoarthrosis, directly translated as “false joint”, is the failure in arthrodesis of an intended spinal fusion. Pseudoarthrosis may occur as a result of increased mechanical stress, typically at the caudal end of fusion constructs, leading to gradual loss of the bone graft, failure of bone graft incorporation, and hardware failure [84,85]. Increased number of vertebral segments in fusion surgery is also associated with higher odds of pseudoarthrosis [86].

Risk factors that increase susceptibility to complications of spine surgery related to instrumentation have been described. Patient-related risk factors common to these complications include increasing age, obesity, history of smoking and hypertension, immunosuppression, greater pelvic incidence, low bone density, and connective tissue dysplasia [77,87,88]. For procedure-related risk factors, hardware failure is associated with posterior subtraction osteotomies and multilevel constructs [77]; PJK is associated with placement of C2 pedicle screws [89]; and ASD is associated with C5/C6 and C6/C7 cervical spine arthrodesis [74]. Prevention and treatment of these complications thereby requires careful consideration of these risk factors, including the management of patient comorbidities and robust surgical planning that include procedure-specific strategies to minimize complication risk.

Complications of spine surgery unrelated to instrumentation include surgical site infection and recurrent disc herniation. Surgical site infections are the most common complications of spine surgery. With 30-day incidence rates reaching 10%, these infections may be superficial or deep, causing morbidity with consequent delayed postoperative discharge [90]. Risk factors predisposing to surgical site infections include obesity, conditions that delay wound healing, such as diabetes, hypertension, and smoking, and prolonged operative times [90,91]. Treatment of surgical site infections typically involves addressing patient-specific risk factors, appropriate antimicrobial therapy, surgical debridement, and sterile wound care [92,93]. Disc herniations can also recur after a clinically symptom-free period post-discectomy. These re-herniations occur at the same level as the previously treated herniation, with greater risk seen in those with specific morphological features, smokers, and diabetics [94,95]. Due to a higher incidence of clinically asymptomatic herniation recurrences, postoperative radiological imaging is the mainstay diagnostic modality, which may guide treatment in the form of conservative measures or revision surgery [96].

Neurological complications after spine surgery can be related or unrelated to instrumentation, typically presenting as radiculopathy. Rates of neurological injury in spinal instrumentation surgeries occur in up to 6% of cases, with trauma to neural elements originating from mispositioning of pedicle screws or compression from graft material, cages, and dura sealing materials [97,98]. The use of image-guided screw placement in addition to intraoperative neuromonitoring techniques have served as techniques to mitigate the risk of neurologic injury following instrumentation [99,100]. Techniques that risk damage to muscle or nerve dissection, such as the psoas muscle and some nerves of the lumbosacral plexus in lateral lumbar interbody fusion (LLIF), are reported to be associated with neurological complications [101]. Posterior cervical fusions are also associated with C5 palsy, which is proposed to be caused by significant spinal cord drift and related changes following chronic compression of the C5 spinal nerve [66]. Neurological injuries post-spine injury may also occur from nerve root compression by space-occupying lesions, such as abscesses [102], hematoma, or recurrent disc herniations, and disrupted blood supply [97,98]. Cervicothoracic spine surgeries, particularly those involving anterior approaches, pose a risk of injuring sympathetic nerves and related structures, leading to complications such as Horner’s syndrome. Preventive strategies include careful identification of sympathetic structures and minimizing traction during surgical exposure [103,104]. Bleeding from the vertebral artery is another significant risk, particularly with atypical entry points into its canal at the levels of C4–C5 [105,106]. Additionally, thoracic duct injury, leading to lymphorrhagia, is a rare, but potential complication during cervicothoracic surgery [107]. Injury to the thoracic duct may lead to chylous leakage, which can be managed by identifying and carefully avoiding the duct intraoperatively. Intraoperative injury can be managed with ligation or use of sealants.

### 4.2. Patient Outcome Metrics

Outcome measures are used in spine surgery patients to assess their clinical status. These measures may be objective, which are quantifiable, unbiased instruments, or subjective, which rely on patient-specific experiences and perspectives. Both objective and subjective measures in spine surgery provide patients with a score or risk categorization, useful in clinical decision making [102].

Subjective outcome metrics, termed patient-reported outcome measures (PROMs), are widely used in spine surgery and can be categorized as generic measures, disease-specific measures, or pain measures (Table 3). Generic PROMS include the item short form health survey (SF-36) [108] and the standardized instrument of the Euroquol Group (EQ-5D) [109] questionnaires, used in assessing patient health-related quality of life (HRQoL). Disease-specific PROMS used in spine surgery include the cervical spine outcomes questionnaire (CSOQ) [110], modified Japanese Orthopedic Association (mJOA) score [111], and the neck disability index (NDI) [112] for cervicothoracic spine disease; the Oswestry Disability Index (ODI) [113] and Roland-Morris Disability Questionnaire (RMDQ) [114] for lumbar spine disease; the Scoliosis Research Society-22 Patient Questionnaire (SRS-22) [115] for adult spinal deformity; the Beck Depression Inventory (BDI) [116]; and Hospital Anxiety and Depression Scale (HADS) [117] for mood disorders. Pain measures used in spine surgery include the Visual Analog Scale (VAS) [118] and the numeric rating scale (NRS) [119]. PROMs have demonstrated great value in standardizing subjective measures to allow comparisons among groups, providing clinicians with context about patients’ health-related experiences. Most PROMs have demonstrated validity, reliability, and consistency across different health states, facilitating their use in the clinical setting [108,120,121,122].

Despite these advantages, PROMs face several limitations. These crude measures are subjective by nature, leading to bias and confounding. PROMs are also “snapshot” measures, providing information at the point at which they are administered, with the potential to fail to capture important information of the patient’s health journey [102]. PROMs are subject to the “disability” paradox, due to patient attitudes and beliefs, where patients with significant disability report good outcomes, or small changes in the health of this patient group are reported as significant changes [123]. Outcome measures may also fail to respond to change adequately, a property defined by the minimal important difference. An example is the large range of clinically significant ODI change scores, reducing its responsiveness to change in the clinical picture [124]. The assumption of measurement invariance by PROMs leads to a “one size fits all” approach in measuring subjective patient outcomes. The validity and reliability of PROMs may be negatively impacted if different groups of individuals answering the questionnaires have different understanding, perspectives, and interpretation of health-related beliefs and behaviors [124]. It has been noted that patients’ perspectives of their physical health may be influenced by external factors, including pain and mental health [125,126]. There is also a mismatch in the dimensionality of outcome measures and the outcomes to which they are applied. An example is the unidimensional nature of low back pain, which is measured by the ODI, a measuring instrument that is multidimensional in nature, accounting for both physical and psychosocial functioning [127]. This difference in dimensionality predisposes outcome measures by the ODI for low back pain to suffer from confounding effects in psychosocial functioning [128]. This effect is also seen in the use of the NDI for assessing neck disability, with experts recommending screening of major depressive disorder to control for confounding [129]. Ceiling and flooring effects are seen with outcome measures, with the ODI and RMDQ exhibiting complementary properties. The ODI has a small ceiling but large floor effect, resulting in greater sensitivity to change in patients with more severe symptoms. The opposite is true for the RMDQ, which has a small floor but large ceiling effect. Consequently, the RMDQ is more suitable for monitoring patients with milder disease, allowing its complementary use with the ODI in spine patients [130]. The significant floor and ceiling effects of these measures limit their widespread applicability to different phenotypes of spine surgery patients, such as spinal trauma patients [131].

Limitations of subjective outcome measures in spine surgery can be addressed by the design of validated metrics that are specific to different patient populations. Concurrent use of objective measures with PROMs can provide comprehensive assessments of patient outcomes. Examples of objective measures used in spine surgery include daily step count, walking speed, and accelerometry measures [102,132,133]. To address bias associated with PROMs, artificial intelligence algorithms are being incorporated into phone applications, body tracking devices, and wearable sensor systems to capture objective outcome measures in spine surgery [134,135]. AI algorithms also have a place in predicting PROM outcomes, serving as complementary tools in the clinical care of spine surgery patients [136].

### 4.3. Patient Selection

Appropriate patient selection is critical to the success of spine surgery, minimizing complications and improving physical function [137]. However, selecting patients for spine surgery is not always straightforward, due to patient-related factors, surgery-related factors, the lack of consensus in patient selection criteria in some cases, or a combination of these factors (Table 4).

Despite the established advantages of minimally invasive spine surgery (MISS), patient selection criteria are lacking. Patient-related factors affecting appropriate patient selection in MISS include patient comorbidity, particularly multilevel spine disease, the subjective nature of painful pathology, and patient-specific anatomical complexities. The transitional L5/S1 anatomy has been shown to drive patient selection in endoscopic approaches, with sex-related differences in pelvic anatomy leading to increased feasibility of supra-iliac transforaminal approaches in women [138]. Anatomical complexity also impacts surgical planning, either through complicating access to the spine or obscuring anatomic landmarks, which directly makes surgeon skill level or available equipment limiting factors in patient selection [139]. To address variance in patient criteria for MISS, surgery is typically reserved for patients who have tried conservative measures, if appropriate, and those with appropriate physical examination and imaging findings. Despite these measures, accurate patient selection may not always be feasible. For example, contradictory imaging and intraoperative findings have been reported in patients with lumbar stenosis undergoing decompression, where either compression reported on preoperative radiological imaging was absent in direct endoscopic visualization, or compression visualized intraoperatively was missed preoperatively [140]. Thus, a thorough collaborative effort that involves clinical expertise, consideration of patient comorbidity and anatomy, and regular preoperative imaging is warranted in developing comprehensive patient criteria, adherence to which is paramount to favorable patient outcomes [137]. Predictive algorithms for appropriate patient selection have been developed for MISS, including the two minimally invasive surgery deformity algorithms (MISDEF and MISDEF2) for the treatment of spinal deformity [141,142], an image-based patient stratification for endoscopic transforaminal, interlaminar, and translaminar decompression of lumbar spinal stenosis [139], and the FAPDIS algorithm (Facet angle, Anterior pathology, Posterior pathology, Dorsal, Inferior, and Superior migration), which selects patients with compressive pathology for endoscopic decompression [143].

Difficulties in appropriate patient selection in spine surgery can also be seen in the surgical treatment of degenerative spondylolisthesis. The efficacy of surgical treatment in degenerative spondylolisthesis is documented in patients with refractory symptoms despite conservative treatment [144]. However, the choice of patients in whom decompression alone or decompression with fusion is most appropriate is a matter of debate due to the lack of standardized treatment guidelines. Decompression alone has been demonstrated to be superior to conservative management, with treated patients reporting better SF-36 scores, ODI scores, greater improvements in leg and back pain, and good-to-excellent outcomes [145]. Other studies have shown non-inferiority between decompression alone and decompression with fusion in terms of clinical effectiveness [146,147,148], with one retrospective study demonstrating the superiority of decompression alone to decompression with fusion due to reduced blood loss and operative time [149]. Patient-related factors that correlate with optimum success following decompression alone have been described, including elderly age (≥65 years old), predominance of leg pain over back pain, male gender, symptom duration ≤3 months, a lower baseline ODI, and concurrent lumbar disc herniation and stenosis [150]. Notably, elderly patients are deemed more suitable due to the reduced operative time and less morbidity associated with decompression alone, and males are associated with a higher rate of treatment success due to the predisposition of females to abnormal mechanical stress in adjacent vertebrae due to their more stable lumbosacral joint [151]. The criterion of leg pain predominating back pain for the suitability of decompression alone in spondylolisthesis is demonstrated by a preoperative score by Dimitriou et al. [152], which predicts treatment failure in patients with low back pain, in addition to facet joint effusion, tobacco smoking, younger age, previous spine surgery at the same level, and fatty infiltration of paraspinal muscles. Appropriate patient selection for decompression alone is pertinent due to the adverse effects of decompression surgery on the segment’s biomechanics, leading to instability and increased risk of same-segment disease, requiring reoperation [153,154,155]. Supplemental fusion of the segment, with or without instrumentation, has shown value in treating spondylolisthesis, especially where adjacent segment disease or pseudoarthrosis is present [150]. Decompression with fusion is also reported to reduce the risk of pseudoarthrosis [156]. Despite some studies reporting the superiority of decompression with fusion, recent studies have demonstrated comparable, or worse, efficacy of decompression with fusion compared to decompression alone for low-grade spondylolisthesis, using the VAS, mJOA, SF-36, and ODI scores as endpoint measures [157,158,159]. Decompression with fusion is associated with greater morbidity and healthcare costs, longer postsurgical recovery, and risk of adjacent segment degeneration, emphasizing the importance of appropriate patient selection and clinical decision making [160,161].

Addressing the challenge of appropriate patient selection in spine surgery requires a multifaceted approach that integrates advancements in diagnostic technology, surgical techniques, and patient-specific factors. Utilizing high-resolution radiographic imaging in patient assessment will provide a more detailed understanding of the patient’s spinal pathoanatomy. Integration of AI and ML, both in imaging and broader health-related data, will enable identification of subtle pathologies, predicting surgery outcomes, and optimizing patient selection criteria [53,162]. Comprehensive preoperative assessments that evaluate age-specific physical function and activities of daily living, and provide improved understanding of painful symptomatology, will ensure a holistic, yet individualized approach to the patient’s condition and suitability for surgery. In the history and physical examination of pain, the character of the pain is important. Additionally, exacerbating and relieving factors should be queried, as well as tested through a series of motions (e.g., forward flexion, alternating lateral flexion, and extension) and maneuvers (e.g., axial and mechanical loading) [163]. These factors should be incorporated in establishing consensus guidelines based on high-quality research that leverages interdisciplinary collaborations from neurosurgery, orthopedic surgery, radiology, and pain management teams.

## 5. Future Directions

The future of spine surgery lies in the intersection of technological innovation, personalized patient care, and enhanced surgical education. Addressing the anatomical and technical challenges requires a continued focus on the development of more intuitive surgical technologies, such as augmented reality and robotics, that can provide real-time anatomical guidance [30,164]. The integration of artificial intelligence in preoperative planning and intraoperative decision making holds the potential to significantly reduce surgical errors and improve patient outcomes. Moreover, the creation of comprehensive databases for spinal pathologies can aid in the development of more accurate and generalizable AI models [165].

In terms of patient-centered care, there is a need for the development of more reliable and patient-specific outcome measures that can accurately capture the impact of surgery on quality of life. Furthermore, enhancing patient selection through predictive modeling and a better understanding of the factors that contribute to successful outcomes will be crucial [166,167].

Finally, addressing the learning curve associated with spine surgery demands an emphasis on surgical education, with a focus on simulation-based training and competency-based learning models. This will ensure that surgeons are well equipped to handle the complexities of spinal surgery, ultimately leading to safer and more effective patient care [25].

## 6. Conclusions

The continuous evolution of spine surgery presents significant challenges that necessitate a multifaceted and patient-centered approach. Advances in surgical techniques, the integration of emerging technologies, and a deeper understanding of spinal anatomy have substantially improved patient outcomes. However, these advancements also introduce new complexities, including the technical intricacies of modern surgical approaches, the variability of spinal anatomy, and the integration of artificial intelligence into the surgical workflow. Patient-centered issues, such as the management of postoperative complications, the selection of appropriate outcome metrics, and patient selection criteria, remain critical for ensuring surgical success. Addressing these challenges requires a commitment to continual education and training for surgeons, the development of intuitive surgical technologies, and the enhancement of patient-specific outcome measures. The future of spine surgery lies in embracing these advancements while prioritizing patient safety and outcomes through research and development, emphasizing the importance of a comprehensive approach that leverages technological innovations, personalized patient care, and advanced surgical education.

## Figures and Tables

**Table 1:** Complexity and variability in spinal anatomy, how they present challenges in spine surgery, and recommendations to address these challenges.

Anatomical Region	Complexities	Variabilities	Surgical Challenges	Recommendations
Craniocervical junction (CCJ)	Houses spinal cord, cranial nerves IX–XII, and vertebral artery.	Atlantooccipital assimilation, basilar invagination, high-riding vertebral arteries.	Limited landmarks and visualization; close proximity to critical structures.	Thorough preoperative imaging; mastery of local anatomy.
Cervical spine	Instrumentation near the vertebral artery.	Anatomical variants and previous surgeries.	Low margin of error due to close apposition to vertebral artery.	Tailored surgical approaches; posterior subtraction osteotomies often performed at C7.
Cervicothoracic junction (CTJ)	Transitional area with thin and convergent pedicles.	Variations in arthrodesis extent requirements.	Specific techniques needed to prevent failure and adjacent segment diseases.	Careful consideration in instrumentation technique.
Thoracic spine	Smaller pedicles compared to the lumbar spine.	Rib variants may lead to wrong entry points.	Increased risk of spinal cord injury with instrumentation.	Caution with percutaneous screws, attention to anatomical variations.
Lumbosacral junction (L5-S1)	Challenges in surgical access and visualization.	Large sacral ala, high-riding ilium, lateral-lying facet joints.	Complications with nerve root trauma from blind removal of disc fragments.	Larger surgical corridors; minimize blind technique use.

**Table 2:** Summary of postoperative complications in spine surgery, categorized into complications related to instrumentation and non-instrumentation complications.

Type of Complication	Incidence Rate	Relevance
**Complications related to Instrumentation**		
Dural tears	1–17%, higher in revision surgeries	Instrumentation complications increase with age, previous surgeries, and poor bone quality.
ASD	2.9% (cervical), 2.5% (lumbar)	
Hardware failure	2.8–32%	
Junctional kyphosis	5% to 46% (PJK) and 7.1% to 24% (DKJ)	
Pseudoarthrosis	5 to 35% (lumbar)	
**Non-Instrumentation Complications**		
Surgical site infections	Up to 10%	Proper patient selection and advanced imaging help mitigate the risk of these complications.
Recurrent disc herniations	5–18%	
Neurological deficits	Up to 6%	

**Table 3:** Characteristics of patient-reported outcome measures (PROMs) in spine surgery, their limitations, and recommendations for addressing these challenges.

Outcome Measure	Description	Number of Items	Score	Indications	Limitations	Recommendations
***Generic PROMs***						
EQ-5D	Measures health in five dimensions (mobility, self-care, usual activities, pain/discomfort, anxiety/depression) across three levels. A total of 3125 health states are defined.	25	0 (death) to 1.0 (perfect health).	Generic measure of health.	Ceiling effects in general population.	Revise levels of severity to increase reliability and reduce ceiling effects.
SF-36	Evaluates health through eight domains, yielding two summary scores: Physical Component Summary (PCS) and Mental Component Summary (MCS).	36	No single score is produced.	Generic measure of health.	Subjective criteria and variable patient appraisal affect reliability.	Use alongside spine-specific instruments like ODI or NDI to gain detailed insights into spine-related health impacts.
***Cervical Spine PROMs***						
CSOQ	Composite measures include healthcare utilization, symptoms, distress, disability, and pain severity in shoulder/arm and neck.	35	0–100.	Cervical spine surgery.	May not address non-cervical spine issues.	Supplement with additional measures that assess general health or other specific spine areas to provide a comprehensive assessment.
mJOA	Assesses cervical myelopathy in four components. Score ranges from 0–18.	–	0–18; ≥15: mild, 12–14: moderate, <12: severe.	Cervical spondylotic myelopathy.	Requires physician administration and lacks validation.	Combine with objective clinical assessments; validate the scale through extensive clinical trials.
NDI	Ten sections covering pain intensity, personal care, lifting, reading, headaches, concentration, work, driving, sleeping, and recreation.	10	≤7 corresponds to a good outcome.	Neck disability.	Significant floor and ceiling effects; influenced by psychosocial factors.	Screen for depressive disorders; consider multidimensional scoring.
***Lumbar Spine PROMs***						
ODI	Evaluates interference of back pain with daily activities, sleeping, personal care, social life, sex life, and traveling.	10	0–100; 0–20: minimal disability; 21–44: moderate disability; 41–60, severe disability; 61–80, crippling back pain; 81–100, either bed bound or exaggerating symptoms.	Chronic back pain.	Significant floor/ceiling effects, affected by non-pain factors like depression.	Use alongside other tools like RMDQ to cover different disability ranges and incorporate psychological assessment.
RMDQ	Measures activity limitation from lower back pain.	24	0 (no disability) to 24 (maximum disability).	Low back pain.	Large ceiling effect; does not cover psychological or social issues.	Revise to include psychosocial factors and reduce redundancy.
***Mental Health PROMs***						
BDI	Assesses depression over emotional and somatic symptoms.	21	0–63; 0–13, no depression or minimal symptoms; 14–19: mild depression; 20–28: moderate depression; 29–63: severe depression.	Depression, particularly in patients with lower back pain.	Primarily focused on depression, may not address other emotional factors.	Ensure comprehensive use in relevant clinical assessments.
HADS	Measures mood and emotional disorders using anxiety and depression subscales.	14	0–24; >8 in both subscales indicates clinical anxiety or depression.	Used in various health conditions including spine issues.	Not validated for low back pain.	Use together with functional outcome measures (like ODI or NDI); validate for specific conditions like low back pain.
***Scoliosis PROMs***						
SRS-22	Assesses spinal deformity in 4 domains (function/activity, pain, self-perceived image, and mental health) with 2 additional questions about treatment satisfaction.	22	22–110. Higher scores indicate better quality of life.	Spinal deformity.	Influenced by demographic factors.	Adjust scoring to minimize demographic biases.
***Pain PROMs***						
NRS	Rates pain intensity on an 11-point scale.	–	0 (no pain) to 10 (maximum pain).	Back and leg pain.	Does not reflect pain complexity or variability.	Enhance with tools that capture dynamic aspects of pain.
VAS	Simple 100 mm line from no pain to severe pain.	–	0 (no pain) to 10 (worst imaginable pain).	Back and leg pain.	Subjective and may not quantify pain impact on function.	Incorporate questions regarding pain interference with function to capture a more complete picture of pain’s effects on life quality.

**Table 4:** Key factors affecting patient selection in spine surgery, including anatomical complexity, comorbidities, predictive algorithms, and challenges in degenerative spondylolisthesis.

Factors Affecting Patient Selection	Examples	Relevance
Anatomical complexity	Transitional L5/S1 anatomy, pelvic anatomy differences.	Affects choice of endoscopic approaches.
Comorbidities	Obesity, diabetes, multilevel disease.	May preclude patients from certain minimally invasive surgeries.
Predictive algorithms	MISDEF, FAPDIS, image-based stratification.	Assist in selecting appropriate patients for MISS.
Challenges in degenerative spondylolisthesis	Deciding between decompression alone vs. decompression with fusion.	Patient-related factors (age, pain type) affect outcomes.

## Data Availability

Not applicable.
